# Correction: Fusion algorithm of visible and infrared image based on anisotropic diffusion and image enhancement

**DOI:** 10.1371/journal.pone.0249546

**Published:** 2021-03-30

**Authors:** The PLOS ONE Staff

The title is incorrect. The correct title is: Fusion algorithm of visible and infrared image based on anisotropic diffusion and image enhancement. The correct citation is: Huang H, Dong L, Xue Z, Liu X, Hua C (2021) Fusion algorithm of visible and infrared image based on anisotropic diffusion and image enhancement. PLoS ONE 16(2): e0245563. https://doi.org/10.1371/journal.pone.0245563.

The publisher apologizes for the error.
