# Supplementary material for: Fusion algorithm of visible and infrared image based on anisotropic diffusion and image enhancement (capitalize only the first word in a title (or heading), the first word in a subtitle (or subheading), and any proper nouns)
Source: PLoS One. 2021 Feb 19;16(2):e0245563. doi: 10.1371/journal.pone.0245563 (PMC7894873; doi:10.1371/journal.pone.0245563)
Supplement: Attachment — Submitted filename: Response to Reviewers.pdf [file pone.0245563.s001.pdf]

Dear Dr. Raja:

The article " Fusion algorithm of visible and infrared image based on anisotropic diffusion and image enhancement " has been revised in strict accordance with the comments of the review experts. The specific changes are as follows, please edit your review.

Comment 1:

1、 Compute Q AB/F, N AB/F and L AB/F and deduce the experimental interpretation. Gradient based metrics are important to validate your technique.

Modify the description:

The article has added the Q AB/F, L AB/F, MI, and SSIM indicators in Table 12 to Table 15 according to the requirements. Table 16 is Rank score results.The details are as follows:

$Q^{AB/F}$ [53-54] is the important of evaluating the success of gradient information transfer from the inputs to the fused image. $L^{AB/F}$ is a measure of the information lost during the fusion process. Considering the correlation between $Q^{AB/F}$ and $L^{AB/F}$, we only calculate $Q^{AB/F}$ and $L^{AB/F}$ here, but as a supplement, we add two other correlation functions MI[55] and SSIM[56]. MI represents the amount of information the fused image obtains from the input images. SSIM reflects the structural similarity between the input images and the fused image. The quality assessment results are shown from Table 8 to Table 15. We rank the 8 algorithms in order from from high to low according to the results of performance indicators.

Table 12. $Q^{AB/F}$ quality assessment results

Method	Nato camp	Nightstreet	Kaptein	Gun	Rank score
GTF	0.3779	0.3768	0.3404	0.3145	2
PCNN	0.4282	0.5043	0.4504	0.5870	7
DTCWT	0.4319	0.4691	0.4620	0.5640	8
CVT	0.3885	0.4404	0.4271	0.5275	5
MSVD	0.2896	0.2929	0.3168	0.3811	1
GF	0.4518	0.4834	0.3911	0.6079	6
LRR	0.4109	0.4485	0.3812	0.4633	3
Ours	0.3929	0.4854	0.3588	0.5717	**4**

Table 13. $L^{AB/F}$ quality assessment results

Method	Nato camp	Nightstreet	Kaptein	Gun	Rank score
GTF	0.5827	0.5877	0.6211	0.6557	2
PCNN	0.3706	0.3415	0.3265	0.2666	7
DTCWT	0.4900	0.4503	0.4238	0.3126	5
CVT	0.4999	0.4646	0.4155	0.3274	4
MSVD	0.7047	0.6904	0.6662	0.6036	1
GF	0.4188	0.2652	0.4273	0.3414	6
LRR	0.5374	0.5208	0.5869	0.4979	3
Ours	0.1698	0.1638	0.1270	0.1616	**8**

Table 14.MI quality assessment results

Method	Nato camp	Nightstreet	Kaptein	Gun	Rank score
GTF	1.4036	1.9337	2.0082	1.0713	8
PCNN	1.1279	1.2856	1.4142	1.3442	5
DTCWT	1.0248	1.1848	1.2420	1.0047	3
CVT	0.9699	1.0867	1.1610	0.9885	2
MSVD	1.0617	1.5610	1.4100	1.3770	6
GF	1.3601	1.2918	1.9784	1.8904	7
LRR	1.1611	1.2243	1.3596	1.2153	4
Ours	0.9068	1.0387	0.9719	0.9485	**1**

Table 15.SSIM quality assessment results

Method	Nato camp	Nightstreet	Kaptein	Gun	Rank score
GTF	0.6870	0.7438	0.7327	0.3878	3
PCNN	0.7115	0.7478	0.7451	0.5163	7
DTCWT	0.6903	0.6540	0.7646	0.4895	5
CVT	0.6894	0.6535	0.7578	0.4874	4
MSVD	0.7155	0.6786	0.7787	0.4999	6
GF	0.6399	0.6332	0.6745	0.5065	2
LRR	0.7521	0.7657	0.7974	0.5222	8
Ours	0.6074	0.6785	0.6128	0.4795	**1**

The higher the objective index SF, MG, EG, EI, $Q^{AB/F}$, MI, and SSIM the better the fusion effect will be, but $L^{AB/F}$ is not. Combined with the data in Table 8 to Table 11 and Table 13, it can be seen that in the performance of its own image, the algorithm in this paper performs well, has good edge contour characteristics, and the loss of fusion is the least. However, the connection with the source image is slightly weaker. The reason is that after Laplacian fusion, the features and details from different images may be merged, resulting in a change in the feature structure.

[30] D Liu, D Zhou, R Nie, et al. “Infrared and visible image fusion based on convolutional neural network model and saliency detection via hybrid l0-l1 layer decomposition”. Journal of Electronic Imaging, 2018, 27(6): 063036.

[31] R Hou, R Nie, D Zhou, et al. “Infrared and visible images fusion using visual saliency and optimized spiking cortical model in non-subsampled shearlet transform domain”. Multimedia Tools and Applications, 2019, 78(20): 28609-28632.

[49] R Shapley, P Lennie. “Spatial frequency analysis in the visual system”. Annual review of neuroscience, 1985, 8(1): 547-581.

[50] B Pan, Z Lu, H Xie. “Mean intensity gradient: an effective global parameter for quality assessment of the speckle patterns used in digital image correlation”. Optics and Lasers in Engineering, 2010, 48(4): 469-477.

[51] J Gauss, JF Stanton, RJ Bartlett. “Coupled‐cluster open‐shell analytic gradients: Implementation of the direct product decomposition approach in energy gradient calculations”. The Journal of chemical physics, 1991, 95(4): 2623-2638.

[52] X Luo, Z Zhang, C Zhang, et al. “Multi-focus image fusion using HOSVD and edge intensity". Journal of Visual Communication and Image Representation, 2017, 45: 46-61.

[53] C Xydeas, V Petrovi. Objective Image Fusion Performance Measure, Electronics Letters, 2000, 36(4): 308-309.

[54] V Petrovic, C Xydeas. “Objective image fusion performance characterisation”. in:Tenth IEEE International Conference on Computer Vision. IEEE, 2005, 2: 1866-1871.

[55] M Seetha, IV MuraliKrishna, BL Deekshatulu. Data fusion performance analysis based on conventional and wavelet transform techniques. in: IEEE Proceedings on Geoscience and Remote Sensing Symposium, 2005, 4: 2842–2845.

[56] Z Wang, AC Bovik, HR Sheikh, et al. Image quality assessment: from errorvisibility to structural similarity. IEEE Transaction Image Process, 2004, 13(4):600–612.

Comment 2:

2、Use “gun’ Dataset. Gun dataset is captured in night mode contaminated with blur and noise. It is very important how your technique behave in that scenario. Compute proposed and other techniques on “Gun “Dataset.

Modify the description:

The article has added experiments on the 'gun' dataset according to the requirements. The details are as follows:

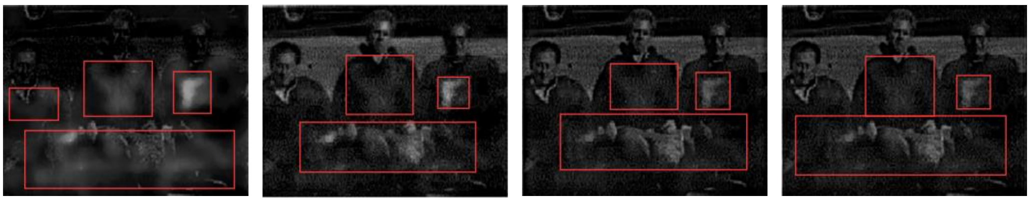

(a) GTF (b)PCNN (c) DTCWT (d) CVT

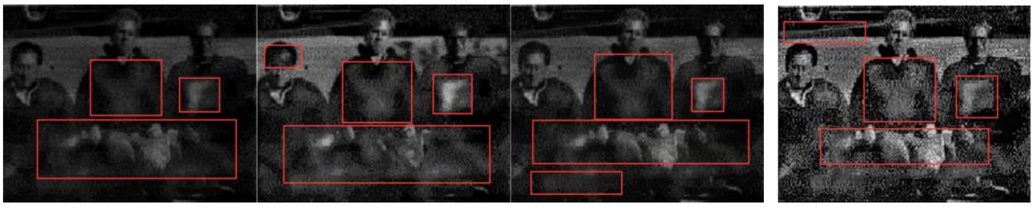

(e) MSVD (f) GF (g) LRR (h) Ours

Fig.14. Fusion results of Gun

The fusion results of Gun are shown in Fig.13. In (a), the infrared target is obvious, but the information of face and background is lost and much artifact are produced. In (b), the fusion results in a "black block" on the face of the right character, and the hand and foot of the character cannot be recognized. In(c) to (e) infrared targets

are relatively fuzzy, and the shadows in the background lose the gradual change characteristics and produce different degrees of artifact. In (f), the infrared target is obvious, but there are different degrees of "black" blocks on the left face and behind the other two people. In (g), only the infrared target of the gun is obvious, and the overall brightness is low. In (h), the infrared target is clear, and the texture after fusion is natura. For example, the shadow in the background can better reflect the real situation of the original scene, which is conducive to human observation.

Comment 3:

3、Redraw figure 1 with smart art or m s visio . This figure seems to be very generic and simple. Kindly draw a more meaningful block diagram.

Modify the description:

The article has revised the fig.1 by m s visio2013 according to the requirements, The details are as follows:

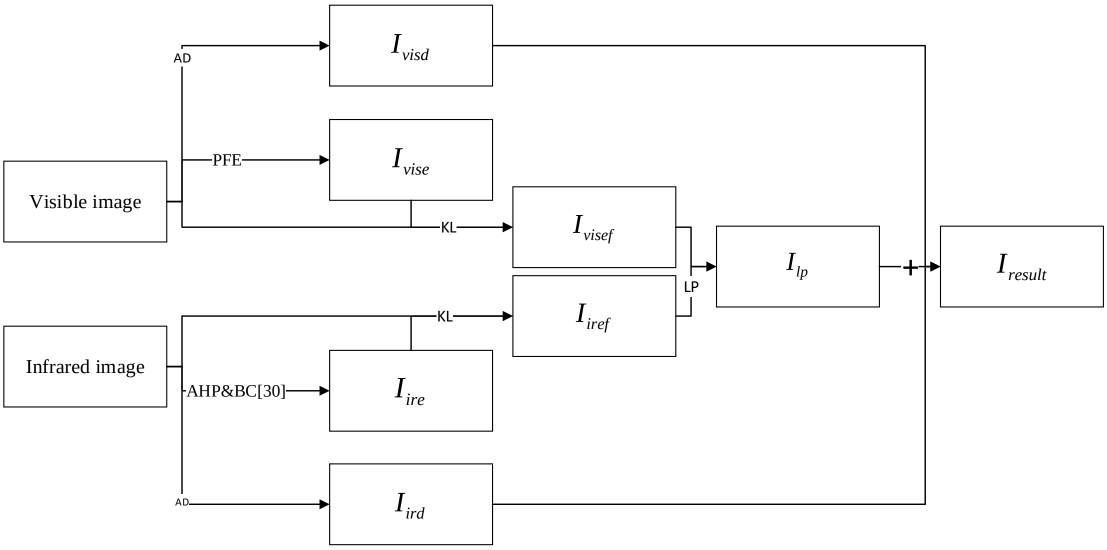

Fig.1. The structure of this paper

Comment 4:

4、Authors have missed to cite the popular and important review articles. There are 4 to 5 important review articles in literature in the field of image fusion. Please cite.

Modify the description:

The article has revised according to the requirements. The details are as follows:

[1] Y Zeng, W Huang, M Liu, et al. Fusion of satellite images in urban area: Assessing the quality of resulting images. in: 2010 18th International Conference on Geoinformatics. IEEE, 2010: 1-4.

[30] D Liu, D Zhou, R Nie, et al. "Infrared and visible image fusion based on convolutional neural network model and saliency detection via hybrid l0-l1 layer decomposition". Journal of Electronic Imaging, 2018, 27(6): 063036.

[31] R Hou, R Nie, D Zhou, et al. "Infrared and visible images fusion using visual saliency and optimized spiking cortical model in non-subsampled shearlet transform domain". Multimedia Tools and Applications, 2019, 78(20): 28609-28632.

[53] C Xydeas, V Petrovi. Objective Image Fusion Performance Measure, Electronics Letters, 2000, 36(4): 308-309.

[54] V Petrovic, C Xydeas. "Objective image fusion performance characterisation". in:Tenth IEEE International Conference on Computer Vision. IEEE, 2005, 2: 1866-1871.

[55] M Seetha, IV MuraliKrishna, BL Deekshatulu. Data fusion performance analysis based on conventional and wavelet transform techniques. in: IEEE Proceedings on Geoscience and Remote Sensing Symposium, 2005, 4: 2842–2845.

[56] Z Wang, AC Bovik, HR Sheikh, et al. Image quality assessment: from errorvisibility to structural similarity. IEEE Transaction Image Process, 2004, 13(4):600–612.

Comment 5：

5、Visual interpretation of results are not very informative. Kindly provide the fine detailing how your architecture is better in preserving the homogeneous and non-homogeneous structures.

Modify the description:

In order to better preserve the homogeneous and non-homogeneous structures, the article has revised the part of the structure according to the requirements.The details are as follows:

(1) The detail layers I_{ird} and I_{visd} of infrared and visible images can be obtained by AD algorithm, to better preserve clear outline and detail information.

(2) We Propose a Power Function Enhancement (PFE) algorithm to simulate illumination for visible images to obtain visible enhanced images I_{vise} , and use Adaptive Histogram Partition (AHP) and Brightness Correction (BC) algorithms to enhance infrared images to obtain infrared enhanced images I_{ire} .

(3) Based on the K-L transform to fuse the enhanced image and the source image, the results I_{iref} and I_{visf} are used as new visible and infrared source image to fully retain the structural characteristics of the image and improve the fusion quality.

(4) In order to better preserve the characteristics of the image and improve the brightness of the image, Laplacian fusion is performed on I_{iref} and I_{visf} to obtain the fused image I_{lp}, and the weighted fusion of I_{lp} , I_{ird} and I_{visd} is used to obtain the result I_{result} .

Comment 6:

6、Abstract and conclusion are not up to mark. Please re – write.

Modify the description:

The article has revised the introduction part according to the requirements. The abstract and conclusion are as follows:

Abstract: Aiming at the situation that the existing visible and infrared images fusion algorithms only focus on highlighting the infrared target, but cannot take into account the characteristics of infrared and visible images. Therefore, it is essential to preserve

the detail of the source images and improve the quality of the source images, this paper proposes an image enhancement fusion algorithm combining Karhunen-Loeve transform and Laplacian pyramid fusion. The detail layer of the source images are obtained by anisotropic diffusion to get more abundant texture information. The infrared images adopt adaptive histogram division and brightness correction enhancement algorithm to highlight thermal radiation targets. A novel power function enhancement algorithm that simulates illumination is proposed for visible images to improve the contrast of visible images and facilitate human observation. In order to improve the fusion quality of images, the source images and the enhanced images are transformed by Karhunen-Loeve to form new visible and infrared images. Laplacian pyramid fusion is performed on the new visible and infrared images, and superimposed with the detail layer images to obtain the fusion result. Experimental results show that the method in this paper is superior to several representative image fusion algorithms in subjective visual effects on public data sets. In terms of objective evaluation, the fusion result performed well on the 8 evaluation indicators, and its own quality was high.

Conclusion:

The effect of image fusion depends on the quality of the fusion algorithm on the one hand, and the quality of the source image on the other. In order to better combine the characteristics of visible and infrared images and obtain more texture information, this paper proposes a new Image enhancement fusion method combining K-L transform and LP fusion. Firstly, the anisotropic diffusion is used to extract the detail layer of the

source image. According to the characteristics of the visible and infrared images, we select different enhancement algorithm. The power function enhancement algorithm is used to simulate the illumination of visible image to improve the brightness of the image and mine the details of the dark image. The infrared image is enhanced by adaptive histogram partition and brightness correction to highlight the characteristics of the target. Secondly, K-L transformation is performed between the enhanced images and the source image to form a new visible and infrared images to ensure that the image is enhanced without distortion and reduce artifact. Finally, LP fusion is performed on the new visible and infrared images, and then the detail layer image is superimposed to obtain the fused image. The experimental results show that the method is subjectively clear texture, high visibility and good observability. In terms of objective indicators, the indicators of the image itself perform well, but the connection with the source image becomes weak. We will conduct further research on this issue in the later period to solve this problem.

Comment 7:

7、Provide mathematics behind the proposed architecture. (If feasible only then).

Modify the description:

The article has added more detailed attribute logic according to the requirements. The details are as follows:

In the third section, we provide the algorithm framework and the mathematical logic behind it. The flow of the algorithm in this paper is as follows:

Algorithm

Input: visible image I_{vis}, Infrared image I_{ir}.

Output: Fused image I_{result}.

1. The detail layers I_{visd} and I_{ird} of infrared and visible image can be obtain by AD algorithm.

Parameters

2. Use the PFE for I_{vis} to get the I_{vise} , Parameters C_1, C_2 . Use the AHP and BC for I_{ir} to get

I_{ire} , Parameters w_1, w_2 , σ_1, w_3 , w_3 , ε_1, $t_{\max}$, N , c_1, c_2 , $\omega_{\max}$, $\omega_{\min}$.

3. The K-L transform between the source image the enhanced image is performed to obtain the enhanced fusion image I_{visef} and I_{iref} .

4. I_{visef} and I_{iref} were fused by LP to obtain I_{lp} , Parameters N_l .

5. Calculate the final fused image I_{result} as $I_{result} = I_{lp} + I_{ird} + I_{visd}$.

Comment 8:

8、There are few linguistic and grammatical errors. Please correct.

Modify the description:

The article has been revised in English as required.

Comment 9:

9、In my view, the novelty of this work is minimum to be accepted by one of the the top journals such as PLOS ONE.

Modify the description:

The structure of the paper has been revised according to the requirements. The details are as follows:

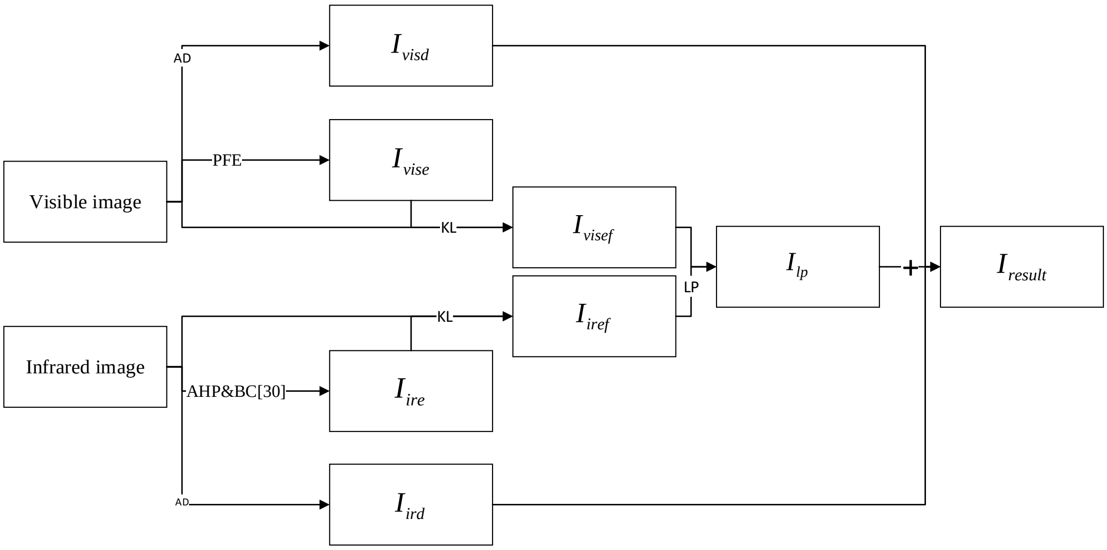

Fig.1. The structure of this paper

Comment 10:

10、This method is almost similar to the work [24]. The main difference is: authors enhanced the contrast of the visible images as a preprocessing step before the fusion.

Modify the description:

The article has revised according to the requirements. The details are as follows:

I am glad you can read my article carefully. Based on your suggestion, I seriously on the framework of the article to be modified. Firstly, we use the anisotropic diffusion (AD) algorithm to obtain the detailed information of the source image. Second, we think that high quality source image is more conducive to image fusion, but because of the different characteristics of visible and infrared images, we use different enhancement algorithms for the two kinds of images. For visible image, we propose a simple and easy Power Function Enhancement (PFE) algorithm to simulate illumination, which is used to improve the brightness of the image and conducive to human observation. For infrared image, we use Adaptive Histogram Partition (AHP) and Brightness Correction (BC) algorithms. Third, in order to ensure the quality of fusion, the enhanced image and the source image for K-L transform. Fourth, we use the Laplacian pyramid to fuse

new visible and infrared images to make the image fusion more coordinated in detail. Finally, superimpose the detail layer and the LP fusion result to obtain the final result. The flow of the algorithm in this paper is as follows:

Algorithm

Input: visible image I_{vis}, Infrared image I_{ir}.

Output: Fused image I_{result}.

1. The detail layers I_{visd} and I_{ird} of infrared and visible image can be obtain by AD algorithm. Parameters

2. Use the PFE for I_{vis} to get the I_{vise}, Parameters C_1, C_2. Use the AHP and BC for I_{ir} to get I_{ire}, Parameters w_1, w_2, σ_1, w_3, w_3, ε_1, $t_{\max}$, N, c_1, c_2, $\omega_{\max}$, $\omega_{\min}$.

3. The K-L transform between the source image the enhanced image is performed to obtain the enhanced fusion image I_{visef} and I_{iref}.

4. I_{visef} and I_{iref} were fused by LP to obtain I_{lp}, Parameters N_l.

5. Calculate the final fused image I_{result} as $I_{result} = I_{lp} + I_{ird} + I_{visd}$.

Comment 11:

11、There are many grammatical errors scattered through out the paper which create the sloppy impression on the paper. Example, section 3.2 heading (3.2 Infrared image fusion base on ddaptive histogram partition and 178 brightness correction).

Modify the description:

The article has been revised in English as required.

Comment 12:

12、Considering the organization of the manuscript, the original proposal is not clearly evidenced. For example, the paper uses Adaptive Histogram Partition (AHP) and Brightness Correction (BC) algorithms to enhance infrared images to highlight the target object. As we know, the infrared images usually consist of thermal radiation characteristics. Please explain the necessity of enhancement of infrared images.

Modify the description:

Description of the necessity of infrared image enhancement. The details are as follows:

The main purpose of infrared image enhancement is to enhance the edge sharpness of the image and improve the fuzzy state of the infrared image. It is more flexible and realistic to stretch the gray level in the Adaptive Histogram Partition. The Brightness Correction uses the moderate gray level of the infrared image, the gray level is more rich, and the expression is enhanced. The results of infrared source image enhancement are as follows:

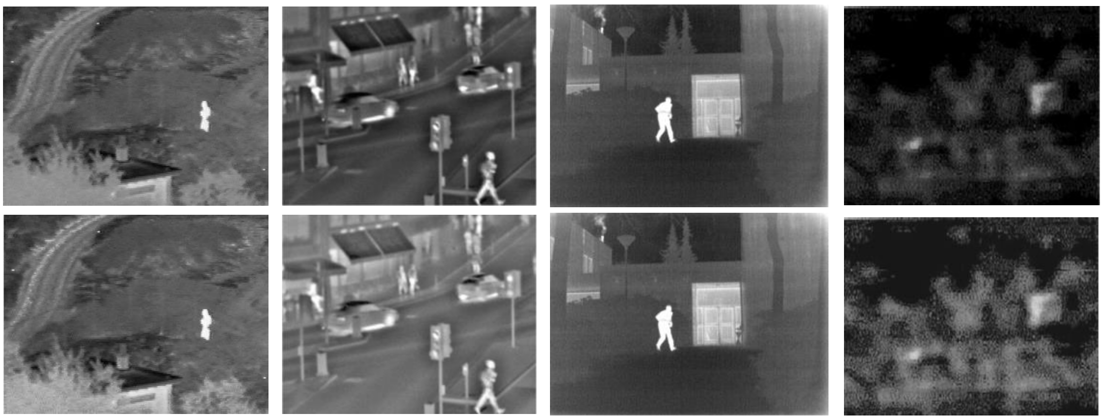

(a) (b) (c) (e)

It can be seen from figures (a) and (c) that the algorithm does not blindly improve the overall brightness of the image, resulting in over enhancement. In figure (b), the brightness of the image is significantly improved, and the infrared target is obvious. In figure (d), the fuzzy state of infrared image is obviously improved, and the gray level of infrared image is more convenient for observation.

Comment 13:

13、 Edge gradient operator (Q AB/F), mutual information and structural similarity (SSIM) are also two important metrics, please add them.

Modify the description:

The article has added the $Q^{AB/F}$, mutual information and SSIM indicators in Table 12,

Table 15 and Table 16 according to the requirements, The details are as follows:

$Q^{AB/F}$ is the important of evaluating the success of gradient information transfer from the inputs to the fused image. MI represents the amount of information the fused image obtains from the input images. SSIM reflects the structural similarity between the input images and the fused image.

Table 12. $Q^{AB/F}$ quality assessment results

Method	Nato camp	Nightstreet	Kaptein	Gun	Rank
GTF	0.3779	0.3768	0.3404	0.3145	2
PCNN	0.4236	0.4327	0.3496	0.3348	3
DTCWT	0.4319	0.4691	0.4620	0.5640	7
CVT	0.3885	0.4404	0.4271	0.5275	5
MSVD	0.2896	0.2929	0.3168	0.3811	1
GF	0.4518	0.4834	0.3911	0.6079	**8**
LRR	0.4109	0.4485	0.3812	0.4633	4
Ours	0.3929	0.4854	0.3588	0.5717	5

Table 14.MI quality assessment results

Method	Nato camp	Nightstreet	Kaptein	Gun	Rank
GTF	1.4036	1.9337	2.0082	1.0713	6
PCNN	3.0891	3.0446	2.2695	2.4144	**8**
DTCWT	1.0248	1.1848	1.2420	1.0047	3
CVT	0.9699	1.0867	1.1610	0.9885	2
MSVD	1.0617	1.5610	1.4100	1.3770	5
GF	1.3601	1.2918	1.9784	1.8904	7
LRR	1.1611	1.2243	1.3596	1.2153	4
Ours	0.9068	1.0387	0.9719	0.9485	1

Table 15.SSIM quality assessment results

Method	Nato camp	Nightstreet	Kaptein	Gun	Rank score
GTF	0.6870	0.7438	0.7327	0.3878	4
PCNN	0.6552	0.6104	0.6474	0.3964	1
DTCWT	0.6903	0.6540	0.7646	0.4895	6
CVT	0.6894	0.6535	0.7578	0.4874	5
MSVD	0.7155	0.6786	0.7787	0.4999	7
GF	0.6399	0.6332	0.6745	0.5065	3
LRR	0.7521	0.7657	0.7974	0.5222	**8**
Ours	0.6074	0.6785	0.6128	0.4795	2

The performance of the algorithm in this paper on these three evaluation indicators

is not as good as expected, because the LP fusion process will change the structure of the image. But in terms of the performance indicators of the image itself, its advantages are obvious, combined with visual experience, it is more suitable for human observation.

Comment 14:

1、In experimental results, I think the brightness and detail of target are not satisfactory.

Modify the description:

The article has made the some modifications in order to better retain the brightness and detail of target according to the requirements, The details are as follows:

In order to better highlight infrared targets and improve image brightness, LP fusion is performed on each spatial frequency layer separately, so that different fusion operators can be used to highlight the features and details of specific frequency bands according to the characteristics and details of different frequency bands of different decomposition layers.

An important property of the LP is that it is a complete image representation: the steps used to construct the pyramid may be reversed to accurately merge the resulting new image, LP fusion results are as follows[35]:

$$I_{lp} = LP_0 + \left(Expand\left(LP_1 + Expand\left(LP_2 + ... + Expand\left(LP_{N_l}\right)\right)\right)\right). \tag{22}$$

Where LP_l is the l-th level image decomposed from LP. and $Expand$ operator is the inverse of $Reduce$ operator.

In the final fusion stage, in order to better retain detailed information, Superimpose the result of LP fusion with the detailed image obtained in the section 2. The final fusion results are defined as follows:

$$I_{result} = I_{lp} + I_{ird} + I_{visd}. \tag{23}$$

Comment 15:

15、Some of the latest literatures should be cited. Infrared and visible images fusion using visual saliency and optimized spiking cortical model in non-subsampled shearlet transform domain,Multimed Tools Appl (2019) 78:28609–28632.Infrared and visible image fusion based on convolutional neural network model and saliency detection via hybrid l0-l1 layer decomposition, J. Electron. Imaging 27(6), 063036 (2018).

Modify the description:

The article has cited latest literatures according to the requirements. The details are as follows:

[30] D Liu, D Zhou, R Nie, et al. “Infrared and visible image fusion based on convolutional neural network model and saliency detection via hybrid l0-l1 layer decomposition”. Journal of Electronic Imaging, 2018, 27(6): 063036.

[31] R Hou, R Nie, D Zhou, et al. “Infrared and visible images fusion using visual saliency and optimized spiking cortical model in non-subsampled shearlet transform domain”. Multimedia Tools and Applications, 2019, 78(20): 28609-28632.
